# Effects and mechanisms of natural alkaloids for prevention and treatment of osteoporosis

**DOI:** 10.3389/fphar.2022.1014173

**Published:** 2022-09-23

**Authors:** Bingfeng Lin, Pingcui Xu, Juan Zheng, Xuehui Deng, Qitao Ye, Zhongping Huang, Nani Wang

**Affiliations:** ^1^ Department of Medicine, Zhejiang Academy of Traditional Chinese Medicine, Hangzhou, China; ^2^ Hangzhou Institute for Food and Drug Control, Hangzhou, China; ^3^ School of Pharmacy, Zhejiang Chinese Medical University, Hangzhou, China; ^4^ College of Chemical Engineering, Zhejiang University of Technology, Hangzhou, China

**Keywords:** alkaloids, osteoporosis, osteoblasts, osteoclasts, NFκB, MAPK

## Abstract

Natural alkaloids are polycyclic, nitrogen-containing, and basic compounds obtained from plants. In this review, the advances in bioactive alkaloids with respect to their chemical structures, herbal sources, and effects for the prevention and treatment of osteoporosis are discussed. Anti-osteoporosis alkaloids are classified into six categories based on the chemical structure, namely, isoquinoline alkaloids, quinolizidine alkaloids, piperidine alkaloids, indole alkaloids, pyrrolizidine alkaloids and steroidal alkaloids. They promote mesenchymal stem cells differentiation, improve osteoblast proliferation, stimulate osteoblast autophagy and suppress osteoclast formation. These natural alkaloids can regulate multiple signaling pathways, including interrupting the tumor necrosis factor receptor associated factor 6- receptor activator of nuclear factor kappa B interaction, inhibiting the nuclear factor kappa B pathway in osteoclasts, activating the p38 mitogen-activated protein kinases pathway in osteoblasts, and triggering the wingless and int-1 pathway in mesenchymal stem cells. This review provides evidence and support for novel drug and clinical treatment of osteoporosis using natural alkaloids.

## Introduction

Osteoporosis has become a major global public health problem, which is manifested by reduced bone strength, leading to an increased risk of fracture (Langdahl, 2021). Many different cell types are involved in the bone remodeling, including cells of the osteoblast lineage and those of the osteoclast lineages (Kenkre and Bassett, 2018). Osteoblasts secrete type I collagen, alkaline phosphatase (ALP) and osteocalcin to form osteoid (Kim et al., 2020). Osteoclasts are derived from the monocyte/macrophage hematopoietic precursors and responsible for the bone resorption (Henriksen et al., 2014). Bone destruction promotes the movement of marrow mesenchymal stem cells (MSCs) to new excavated sites, where they differentiate into osteoblasts and enhance the bone formation (Song et al., 2022). Several pathways play a crucial role in the maintenance of bone homeostasis. For example, calcium signaling controls proliferation, differentiation and transcription in bone cells (Kang J. Y. et al., 2020). Osteoprotegerin (OPG) is primarily synthesized in osteoblasts and inhibits osteoclastogenesis by binding to the receptor activator of nuclear factor kappa B ligand (RANKL) (Ono et al., 2020; Yasuda, 2021) and preventing it from binding to its receptor, receptor activator of nuclear factor kappa B (RANK) (Taylan et al., 2019; Xu et al., 2021). Wingless and int-1(Wnt)/β-catenin signaling regulates the survival of osteoblasts and differentiation of MSCs (Stuerznickel et al., 2021). Additionally, several pathways are involved in the apoptosis of bone cells, such as nuclear factor-kappa B (NFκB), mitogen-activated protein kinases (MAPK), and mammalian targe of rapamycin (mTOR) signaling pathways (Wong et al., 2019). Drugs targeting these signaling pathways may be valuable for preventing or treating osteoporosis.

Calcium and vitamin D are the recommended nutrients for patients with osteoporosis (Kahwati et al., 2018). Pharmacological interventions include antiresorptive agents that inhibit bone resorption and anabolic agents that promote bone formation (Seeman and Martin, 2019). Classical drugs include bisphosphonates, denosumab, strontium, teriparatide, calcitonin and estrogen (Martiniakova et al., 2020). However, the long-term use of these drugs is always accompanied by adverse side effects, such as esophagitis for bisphosphonates (Fan and Wang, 2020), cellulitis for denosumab (Zhang N. et al., 2020), nasal congestion for calcitonin (Zhang et al., 2021) and an increased risk of breast cancer for estrogens (Black and Rosen, 2016). More effective treatment methods for osteoporosis are desirable (Lin et al., 2019; Xie et al., 2021).

Alkaloids represent a large class of nitrogen-containing phytochemicals widely present in medicinal plants (Xie et al., 2021) and food (Rasouli et al., 2020). There are more than ten thousand different natural alkaloids, which can be classified according to their carbon skeletal structures (Ziegler and Facchini, 2008). With the continuous development of modern medication, various biopotentials of natural alkaloids have been explored, including antioxidant (Badaoui et al., 2021), antiproliferative (Hu et al., 2018), anti-obesity (Saunders et al., 2018), antidiabetic (Jan et al., 2018), cardiovascular protection (Li et al., 2021a), anti-aging (Asokan et al., 2018), and anti-inflammatory (Hu et al., 2020) effects in experimental trials and clinical applications. At present, natural alkaloids are the mainstream anti-osteoporosis drugs because of their strong efficacy and low toxicity (Ajebli et al., 2021), and some of them have been used clinically. The main groups of anti-osteoporotic alkaloids include isoquinoline, quinolizidine, piperidine, indole, pyrrolizidine and steroidal alkaloids. This review focuses on studies in the field of natural alkaloids in osteoporosis.

## Anti-osteoporotic effect of alkaloids

### Isoquinoline alkaloids

Isoquinoline alkaloids are derived from tyrosine or phenylalanine (Supplementary Figure S1), and exhibit various bioactivities, such as antitumor, antibacterial, and anti-inflammatory (Shang et al., 2020). Eight isoquinoline alkaloids possess anti-osteoporotic activity, including berberine, tetrahydropalmatine, boldine, tetrandrine, fangchinoline, sinomenine, cepharanthine, and nitidine.

Berberine and tetrahydropalmatine have been isolated from the genus *Coptis* (Li et al., 2019) and *Corydalis* (Ma et al., 2009). Oral administrations of these two compounds inhibit bone loss in many osteoporosis models, such as diabetic mice (Adil et al., 2017; Xie et al., 2018), aged mice (Chen Q. C. et al., 2021), ovariectomized (OVX)-treated rats (He et al., 2017; Zhi et al., 2020) and glucocorticoid-induced mice (Xu et al., 2010) (Table 1). However, berberine undergoes extensive metabolism after oral administration, resulting in low plasma exposure (Fan et al., 2021). Hundreds of berberine derivatives have been prepared and the structure modifications are focused on the C8, C9, C10, C12 and C13 positions. For example, 13-alkyl-substituted berberine showed better anti-inflammation activities than berberine (Zou et al., 2017). The anti-osteoporosis effects and structure-activity relationship of berberine derivatives need to be further clarified.

**TABLE 1:** Applications of natural alkaloids in the osteoporosis treatments.

Alkaloid	Plant source	Animal/cell model	Dose and mode of administration	Index	References
Berberine	Coptidis. genus	diabetic, aged, OVX and glucocorticoid-induced models; MSCs、MC3T3-E1 cells; BMMs induced by RANKL	i.g. 20-100 mg/kg; Cell 0.05-30 μM	Malondialdehyde↓, Superoxide dismutase↑	Xu et al., (2010); Adil et al., (2017); He et al., (2017); Xie et al., (2018); Chen et al., (2021)
Tetrahydropalmatine	Corydalis genus	OVX-induced models; BMMs induced by RANKL	i.p. 4 mg/kg; Cell 4.75-19.00 μM	tumor necrosis factor (TNF)-α↓, interleukin-6 (IL-6)↓, type I collagen C-terminal peptide (CTX-1)↓, tartrate-resistant acid phosphatase 5b (TRACP5b)↓	Zhi et al., (2020)
Boldine	Peumus genus	OVX-induced models; BMMs induced by RANKL	i.g. 20 mg/kg; Cell 25-75 μM	CTX-1↓	Chen et al., (2018)
Tetrandrine	Stephania tetrandra S.Moore	OVX, sciatic-neurectomized, and titanium particle-induced models; BMMs induced by RANKL	i.p. 30-60 mg/kg; Cell 0.25-1 μM	interleukin-1α (IL-1α)↓, interleukin-1β (IL-1β)↓, IL-6↓, TNF-α↓, CTX-1↓, TRAP5b↓, [Ca^2+^]_i_↓	Takahashi et al., (2012); Liu et al., (2020a); Zhong et al., (2020)
Fangchinoline	Stephania tetrandra S.Moore	OVX and prednisolone-induced models; BMMs induced by RANKL	i.p. 5-10 mg/kg; Cell 0.25-1 μM	caspase-3↓, B-cell lymphoma-2↑, microtubule-associated protein 1 light chain 3↑, autophagy-related gene-5↑, Beclin-1↑	(Zhu et al., 2019; Zhou et al., 2020a)
Sinomenine	Sinomenium acutum (Thunb.) Rehder and E.H.Wilson	mycobacterium tuberculosis H37Ra-induced model; MC3T3-E1 cells; BMMs induced by RANKL	i.p. 80-150 mg/kg; Cell 0.1-1 μM	TRACP5b↓, RANKL↓, OPG↑, osteocalcin↑, ALP↑, collagen type I alpha 1↑, osteopontin↑, [Ca^2+^]_i_↓	Li et al., (2013); He et al., (2016)
Lycorine	Amaryllidaceae family	OVX and wear particle-induced model; BMMs induced by RANKL	i.p. 2.5 mg/kg; Cell 0.1-0.4 μM	p-P38↓	Chen et al., (2015)
Cepharanthine	Stephania abyssinica (Quart.-Dill. and A.Rich.) Walp	OVX-induced models; BMMs induced by RANKL	i.p. 20 mg/kg; Cell 0.0625-1 μM	nuclear factor of activated T cells c1 (NFATc1)↓	Zhou et al., (2018)
Nitidine	Zanthoxylum nitidum (Roxb.) DC.	OVX-induced models; BMMs induced by RANKL	i.p. 6 mg/kg;Cell (0.125-1 μM)	NFATc1↓	Liu et al., (2016)
Piperine	Piperaceae family	RAW 264.7 macrophages induced by RANKL and breast cancer cells	Cell 5-100 μM	ALP↑	Deepak et al., (2015)
Arecoline	Areca catechu L	LPS-induced models; MC3T3-E1 cells; BMMs induced by M-CSF or RANKL	i.g. 10 mg/kg; Cell 25-100 μM	ALP↑	Liu et al., (2020a)
Matrine	Sophora flavescens Aiton	OVX-induced models; BMMs induced by RANKL	i.p. 150 mg/kg; Cell 1-4 μM	IL-6↓, TNF-α↓, TRACP5b↓	Chen et al., (2017)
Oxymatrine	Sophora flavescens Aiton	OVX-induced models; BMMs induced by RANKL	i.p. 10 mg/kg; Cell 100-400 μM	CTX-1↓	Jiang et al., (2021)
Aloperine	Sophora genus	OVX-induced models; BMMs induced by RANKL	i.p. 30 mg/kg; Cell 10-50 μM	NFκB↓, extracellular signal-regulated kinases (ERK)↓, c-Jun NH2-terminal kinase (JNK)↓	Hu et al., (2021)
Cytisine	Leguminosae family	OVX-induced models; BMMs induced by RANKL	i.p. 25 mg/kg; Cell 12.5-25 μM	NFATc1↓	Qian et al., (2020)
Harmine	Peganum harmala L	OVX-induced models; RAW264.7 cells induced by RANKL	i.g. 10 mg/kg; Cell 0.3-3 μM	platelet-derived growth factor-BB↑, Type H vessel↑	Huang et al., (2018)
Vindoline	Catharanthus roseus (L.) G. Don	OVX-induced models; BMMs induced by RANKL	i.p. 10 mg/kg; Cell 2.5-10 μM	reactive oxygen↓	Zhan et al., (2020)
Rutaecarpine	Acronychia acronychioides (F. Muell.) T. G. Hartley	OVX-induced models; RAW264.7 cells induced by RANKL	i.g. 5 mg/kg; Cell 1-10 μM	OPG↑, ALP↑, CTX-1↓	Li et al., (2021b)
Neotuberostemonine	Stemona tuberosa Lour	BMMs cells induced by RANKL or cancer cells	Cell 10-50 μM	NFκB↓	Yun et al., (2019)
Stachydrine	Leonurus japonicus Houtt	OVX and LPS-induced model; BMMs induced by RANKL	i.g. 10-22.5 mg/kg; Cell 10-50 μM	NFκB↓, p38↓	Meng et al., (2019); Chen et al., (2021a)
Tomatidine	Green tomato	OVX-induced models; BMMs induced by RANKL	i.p. 30 mg/kg; Cell 2-8 µM	RANK↓	Hu et al., (2019); Liu et al., (2020a)

Boldine, an aporphine alkaloid from the genus *Peumus* (O'Brien et al., 2006), protects against OVX-induced osteoporosis by downregulating bone resorption without affecting bone formation (Chen et al., 2018). Tetrandrine (Luan et al., 2020) and fangchinoline (Merarchi et al., 2018), two benzylisoquinoline alkaloids derived from *Stephania tetrandra* S. More, exhibit notable therapeutic effects in OVX (Zhou L. et al., 2020; Zhong et al., 2020), sciatic-neurectomized (Takahashi et al., 2012), titanium particle (Liu Z. et al., 2020), and prednisolone-induced models (Zhu et al., 2019). Fangchinoline differs from tetrandrine in one side chain of its isoquinoline ring. This minor structural difference leads to a considerable difference in their anti-inflammatory effects. Specifically, tetrandrine had good suppression on murine interleukin-5 and human interleukin-6, but fangchinoline only on human interleukin-6 (Choi et al., 2000). Nevertheless, the differences of their anti-osteoporosis effects remain unclear.

Sinomenine is an emetine isoquinoline alkaloids derived from *Sinomenium acutum* (Thunb.) Rehder and E.H.Wilson, which attenuated bone loss in a *Mycobacterium tuberculosis* H37Ra-induced model (Li et al., 2013). Lycorine, a phenanthridine alkaloids from the *Amaryllidaceae* family (Roy et al., 2018), can protect against OVX-induced and wear particle-induced osteolysis (Chen et al., 2015). Cepharanthine from *Stephania abyssinica* (quart.-dill. and A. Rich.) Walp. (Bailly, 2019) and nitidine from *Zanthoxylum nitidum* (Roxb.) DC. (Khan et al., 2018) could prevent bone loss in OVX-induced model by reducing osteoclast differentiation (Liu et al., 2016; Zhou et al., 2018).

### Piperidine alkaloids

Piperidine alkaloids possess a characteristic saturated heterocyclic ring structure (Green et al., 2012). Piperine is isolated from the *Piperaceae* family (Haq et al., 2021), and alleviates bone resorption in RANKL-induced RAW 264.7 (Deepak et al., 2015). Arecoline from *Areca catechu* L. (Volgin et al., 2019) reduced osteoclastic tartrate-resistant acid phosphatase (TRAP) activity and differentiation of macrophage colony-stimulating factor (M-CSF)/RANKL-induced bone marrow monocytes (BMMs) and lipopolysaccharide (LPS)-induced mice (Liu F. L. et al., 2020).

### Quinolizidine alkaloids

Quinolizidine alkaloids are chemically divided into matrine-type and aloperine-type alkaloids (Wang et al., 2019; Li Y. et al., 2020). Matrine and oxymatrine belong to the matrine-type and are derived from *Sophora flavescens* Aiton (Wang et al., 2019). Matrine suppresses osteoclastogenesis in OVX mice (Chen et al., 2017). Oxymatrine attenuates osteoclast formation in RANKL-induced BMMs and OVX-induced mice (Jiang et al., 2021). Neither of them has an effect on bone formation, indicating that they improve osteoporosis mainly by inhibiting osteoclastogenesis. Aloperine is found in the genus *Sophora* (Zhou H. et al., 2020). It attenuates osteoporosis in OVX-treated mice by inhibiting osteoclastogenesis (Hu et al., 2021). Cytisine is isolated from the *Leguminosae* family and can attenuate bone loss in OVX-treated mice (Qian et al., 2020).

### Indole alkaloids

Indole alkaloids originate from condensation of tryptophan and a terpene moiety (Matsuura et al., 2014). Three anti-osteoporotic compounds have been identified in this class. Vindoline from *Catharanthus roseus* (L.) G. Don, and rutaecarpine from *Acronychia acronychioides* (F.Muell.) Hartley (Tian et al., 2019) have inhibitory effects on osteoclast differentiation from BMMs and mature osteoclastic bone resorption (Zhan et al., 2020; Li et al., 2021b). Notably, harmine from *Peganum harmala* L. (Zhang et al., 2020a; Huang et al., 2021) prevented bone loss by enhancing type H vessel formation and decreasing fat cells in the femora of OVX-induced mice (Huang et al., 2018). The structure-activity relationship analysis showed that the C3–C4 double bond and 7-methoxy group of harmine are crucial for its suppression on osteoclast differentiation (Yonezawa et al., 2011).

### Pyrrolizidine alkaloids

Pyrrolizidine alkaloids are structurally characterized by a 1-azabicyclo azaoctane or aza-bridged pentalene skeleton (Robertson and Stevens, 2014). These alkaloids are secondary metabolites of the genus *Senecio*, *Crotalaria*, *Echium*, and *Eupatorim* (Brugnerotto et al., 2021). Neotuberostemonine, a pyrrolizidine alkaloid derived from *Stemona tuberosa* Lour.*,* can inhibit osteoclastogenesis induced by RANKL (Yun et al., 2019). Stachydrine is the major bioactive component of *Leonurus japonicus* Houtt. (Cheng et al., 2020). It inhibited bone resorption in RANKL-induced osteoclasts, OVX-treated mice (Chen M. et al., 2021), and LPS-induced mice (Meng et al., 2019).

### Steroidal alkaloids

Steroidal alkaloids have a basic steroidal skeleton with a nitrogen atom incorporated as an integral part of the compound (Jiang et al., 2016). They are generally found in the glycoalkaloid form (Dey et al., 2019) and exert various pharmacological properties such as anti-inflammatory, anti-cancer, anti-microbial, and analgesic activities (Jiang et al., 2016). Tomatidine is abundant in the skin of unrip green tomatoes (Bailly, 2021) and can be used to treat osteoporosis. It can attenuate IL-1β-induced degradation of collagen-II by inhibiting NFκB and MAPK signaling in articular chondrocytes and osteoarthritic rats. Tomatidine also improves OVX-induced osteoporosis by suppressing osteoclastogenesis (Hu et al., 2019).

## Anti-osteoporotic mechanisms of alkaloids on osteoclasts

### Receptor associated factor 6- receptor activator of NFκB

RANKL is the key regulator of osteoclast differentiation and activity (Kim et al., 2020). It is a TNF related ligand generated on the surface membrane of osteoblasts (Epsley et al., 2021) that functions as an agonistic ligand for the receptor (RANK) (Nagy and Penninger, 2015). It has been proved that RANKL-knockout mice were protected from bone loss during arthritis, suggesting that osteoclastic bone resorption under inflammatory conditions is dependent on RANKL-RANK signaling (Tsukasaki et al., 2017). Previous reports showed that the treatment of several natural alkaloids can inhibit the RANKL synthesis or interrupt the interaction between RANKL and RANK *in vitro* and *in vivo*, such as sinomenine (Li et al., 2013; Zhou et al., 2017), arecoline (Liu F. L. et al., 2020), and oxymatrine (Jiang et al., 2018; Jiang et al., 2021). In osteoclasts, RANKL-RANK signaling stimulation activates tumor necrosis factor receptor-associated factor 6 (TRAF6) (Park et al., 2017), thereby upregulating NFκB (Walsh et al., 2015) and MAPK (Shi and Sun, 2018), and expression of c-Fos (Takayanagi, 2005). These factors initially induce NFATc1, which is the master regulator of osteoclastogenic genes (Oh et al., 2021). During osteoclast differentiation, TRAF6 is a dominant adaptor of RANK that assembles signaling proteins that direct osteoclast-specific gene expression resulting in differentiation and activation (Ikeda and Takeshita, 2016). Thus, the excessive interaction of RANK with TRAF6 contributes to the elevated resorption activity of osteoclasts during the development of osteoporosis. Tetrahydropalmatine decreases osteoclastogenesis by blocking the RANK-TRAF6 interaction (Zhi et al., 2020) in BMMs and RAW264.7 cells. Tomatidine (Hu et al., 2019) and neotuberostemonine block TRAF6 and NFκB activation and impair the formation of F-actin ring structure in RANKL-induced osteoclasts (Yun et al., 2019). Cytisine attenuates bone loss by inhibiting the v-akt murine thymoma viral oncogene homolog 1 (AKT)-NFATC1 pathway and RANKL-induced association of RANK-TRAF6 in OVX-treated mice (Qian et al., 2020).

### NFκB and MAPK

In addition to RANKL-RANK signaling, inflammatory stimuli (IL-1, IL-6, lipopolysaccharide, TNF-α, etc.) increase osteoclast activation and survival through the NFκB (Zhao et al., 2021) and MAPK pathways (Liao et al., 2021). Indeed, increased levels of inflammatory cytokines are relevant characteristics of primary and secondary osteoporosis (Locantore et al., 2020; Ratajczak et al., 2020). Several chronic inflammatory diseases exhibit a detrimental effect on bone homostasis, such as inflammatory bowel disease (Wu et al., 2020), arthritis (Adami and Saag, 2019) and persistent skin inflammation (Mizutani et al., 2020). For instance, high contents of pro-inflammatory cytokines have been showed to be closely associated with osteoclast-mediated focal bone resorption (Coury et al., 2019). Additionally, postmenopausal women always display a chronic inflammatory phenotype with increased levels of circulating inflammatory factors, which contributes to impaired bone formation during menopause (Fischer et al., 2018). Proinflammatory cytokines are also remarkably elevated during senescence and stimulate osteoclast function during the development of senile osteoporosis (De Martinis et al., 2006). Thus, inflammation may be a pivotal target for the development of effective pharmacological interventions. Berberine and tetrahydropalmatine suppressed bone resorption by decreasing the levels of serum TNF-α and IL-6 in OVX mice (He et al., 2017; Zhi et al., 2020). Berberine inhibits osteoclast activity by suppressing NFκB and AKT signaling (Hu et al., 2008). Tomatidine (Hu et al., 2019), tetrandrine (Zhong et al., 2020) and fangchinoline (Zhou L. et al., 2020) can inhibit osteoclastogenesis and decrease TNF-α, IL-6 and the RANKL/OPG ratio by suppressing NFκB and MAPK signaling. Lycorine protected against osteolysis by inhibiting MAPK signaling in OVX-induced and wear particle-induced models (Chen et al., 2015). Aloperine (Hu et al., 2021) and matrine (Jiang et al., 2021) attenuated inflammation and abrogated RANKL-induced stimulation of the NFκB, AKT and MAPK pathways in OVX mice. Stachydrine inhibits the suppression of NFκB and p38 MAPK signaling pathways in RANKL-induced osteoclasts, OVX-treated mice (Chen M. et al., 2021) and LPS-induced mice (Meng et al., 2019).

### NFATc1

NFATc1 is a dominant membrane of the NFAT transcription factor family, which is vital in the immune system during osteoporosis (Gu et al., 2020). Nuclear translocation of NFATc1 promotes microphthalmia-associated transcription factor (MITF) expression (Yang et al., 2019), autoactivates the *Nfatc1* gene, and stimulates the expression of osteoclast-specific genes by collaborating with other transcription factors (Lorenzo, 2017; Chen et al., 2019). The *Nfatc1* gene has been shown to be methylated in over 30% of postmenopausal women, and the *Fos* gene is methylated in 17% of them (Kalkan and Tosun, 2020). Tetrahydropalmatine (Zhi et al., 2020), oxymatrine (Jiang et al., 2021) and cepharanthine (Zhou et al., 2018) reduce osteoclast differentiation by inhibiting NFATc1 activation in OVX-induced models. Harmine (Yonezawa et al., 2011), sinomenine (Zhang Y. et al., 2019) and piperine (Deepak et al., 2015) inhibit the c-Fos/NFATc1 pathway in RANKL-induced RAW 264.7 macrophages. Oxymatrine (Jiang et al., 2021) and arecoline (Liu F. L. et al., 2020) suppressed the c-Fos/NFATc1 pathway in M-CSF/RANKL-induced BMMs and LPS-induced mice.

### Calcium signaling pathway

RANKL induces the oscillatory changes in the intracellular calcium contents, resulting in the calcium/calcineurin-dependent dephosphorylation, NFATc1 activation, and osteoclastic differentiation (Kang M. A. et al., 2020). Tetrandrine treatment can decrease calcium oscillation in RANKL-induced BMMs (Zhong et al., 2020). LPS exposure enhances calcium entry in osteoclasts by stimulating the expression of RNAKL (Zeng et al., 2020). Sinomenine treatment decreased intracellular calcium in LPS-induced osteoclasts (He et al., 2016).

## Anti-osteoporotic mechanisms of alkaloids on osteoblasts and bone marrow mesenchymal stem cells

Osteoblasts are responsible for bone formation and the creation of a physical framework (Hojo and Ohba, 2020). Runt-related transcription factor 2 (Runx2) is an essential transcription factor for osteoblast differentiation and bound to an osteoblast-specific *cis*-regulatory element in the promoter region of the *Bglap* gene (Komori, 2018). Among alkaloids, sinomenine activates the AKT/Runx2 pathway in osteoblasts (Zhang B. et al., 2019). MAPK activation of Runx2 occurs both *in vivo* and *in vitro* (An et al., 2016). In fact, p38 MAPK phosphorylation is enhanced during osteoblast differentiation, whereas inhibition of this pathway impairs pre-osteoblast differentiation and decreases osteoblast markers (Kang J. Y. et al., 2020). Berberine treatment promotes osteoblast differentiation by upregulating the expression of phosphorylated p38 MAPK (Lee et al., 2008). In addition, as a stress-responsive mechanism, autophagy safeguards osteoblasts against oxidative or inflammatory stress by degrading damaged organelles (Li X. et al., 2020). Fangchinoline protects against prednisolone-induced osteoporosis by inducing autophagy and inhibiting apoptosis in osteoblasts (Zhu et al., 2019). Thus, alkaloids could provide a therapeutic effect on bone formation through activation of the p38 MAPK pathway and autophagy in osteoblasts.

Osteoblasts are derived from MSCs. As a common progenitor, lineage commitment of MSCs to osteoblasts plays an important role in maintaining bone homeostasis (Chen et al., 2016). Numerous studies have provided strong evidence for the role of Wnt signaling in facilitating osteogenic differentiation and inhibiting adipogenic differentiation of MSCs (Han et al., 2019). Berberine can promote osteogenic differentiation of MSCs by increasing the transcriptional activity of β-catenin/T-cell factor (Tao et al., 2016).

## Conclusions and future prospects

**FIGURE 1:**
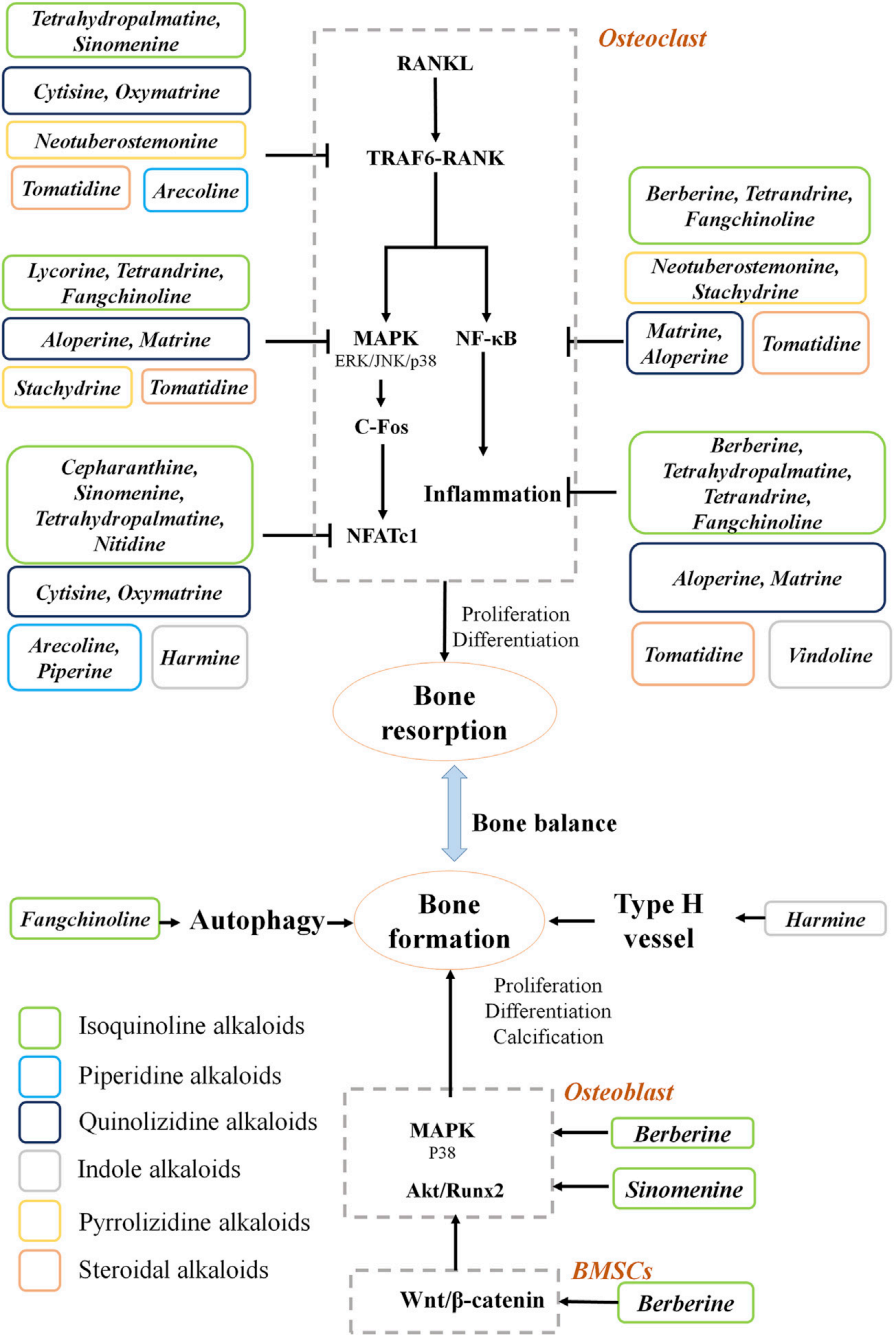
Mechanism of natural alkaloids for preventing and treating osteoporosis.

Pharmacological studies of natural alkaloids on the efficacy and mechanisms of osteoporosis treatment have made remarkable progress. Based on the information collected in this study, the natural alkaloids exert anti-osteoporotic effects mainly by inhibiting bone resorption (Figure 1). Alkaloids reduce osteoclastogenesis through the RANKL-dependent pathways, including TRAF6, NFκB, MAPK, and NFATc1 signaling pathways. The RANKL-independent mechanisms behind the anti-osteoporosis effects of alkaloids remain elusive. Moreover, three main pathways are involved in the osteogenesis promotion effects of the alkaloids. The screening for alkaloids with osteogenic effects should be further carried out. Importantly, the structure-activity relationship investigations need to be performed to provide more evidence for drug development in osteoporosis.

## Abbreviations

AKT, v-akt murine thymoma viral oncogene homolog 1; ALP, alkaline phosphatase; BMMs, bone marrow monocytes cells; CTX-1, type I collagen C-terminal peptide; ERK, extracellular signal-regulated kinases; JNK, c-Jun NH2-terminal kinase; IL-1α, interleukin-1α; IL-1β, interleukin-1β; IL-6, interleukin-6; LPS, lipopolysaccharide; MAPK, mitogen-activated protein kinases; M-CSF, macrophage colony-stimulating factor; MITF, microphthalmia-associated transcription factor; MSCs, marrow mesenchymal stem cells; NFATc1, nuclear factor of activated T cells c1; NFκB, nuclear factor kappa B; OPG, osteoprotegerin; OVX, ovariectomized; RANK, receptor activator of nuclear factor kappa B; RANKL, receptor activator of nuclear factor kappa B ligand; Runx2, runt-related transcription factor 2; TRACP5b, tartrate-resistant acid phosphatase 5b; TNF, tumor necrosis factor; TRAF6, tumor necrosis factor receptor associated factor 6; TRAP, tartrate-resistant acid phosphatase; Wnt, wingless and int-1.
