# Supplementary figures and images for: Effects and mechanisms of natural alkaloids for prevention and treatment of osteoporosis
Source: Front Pharmacol. 2022 Sep 23;13:1014173. doi: 10.3389/fphar.2022.1014173 (PMC9539536; doi:10.3389/fphar.2022.1014173)

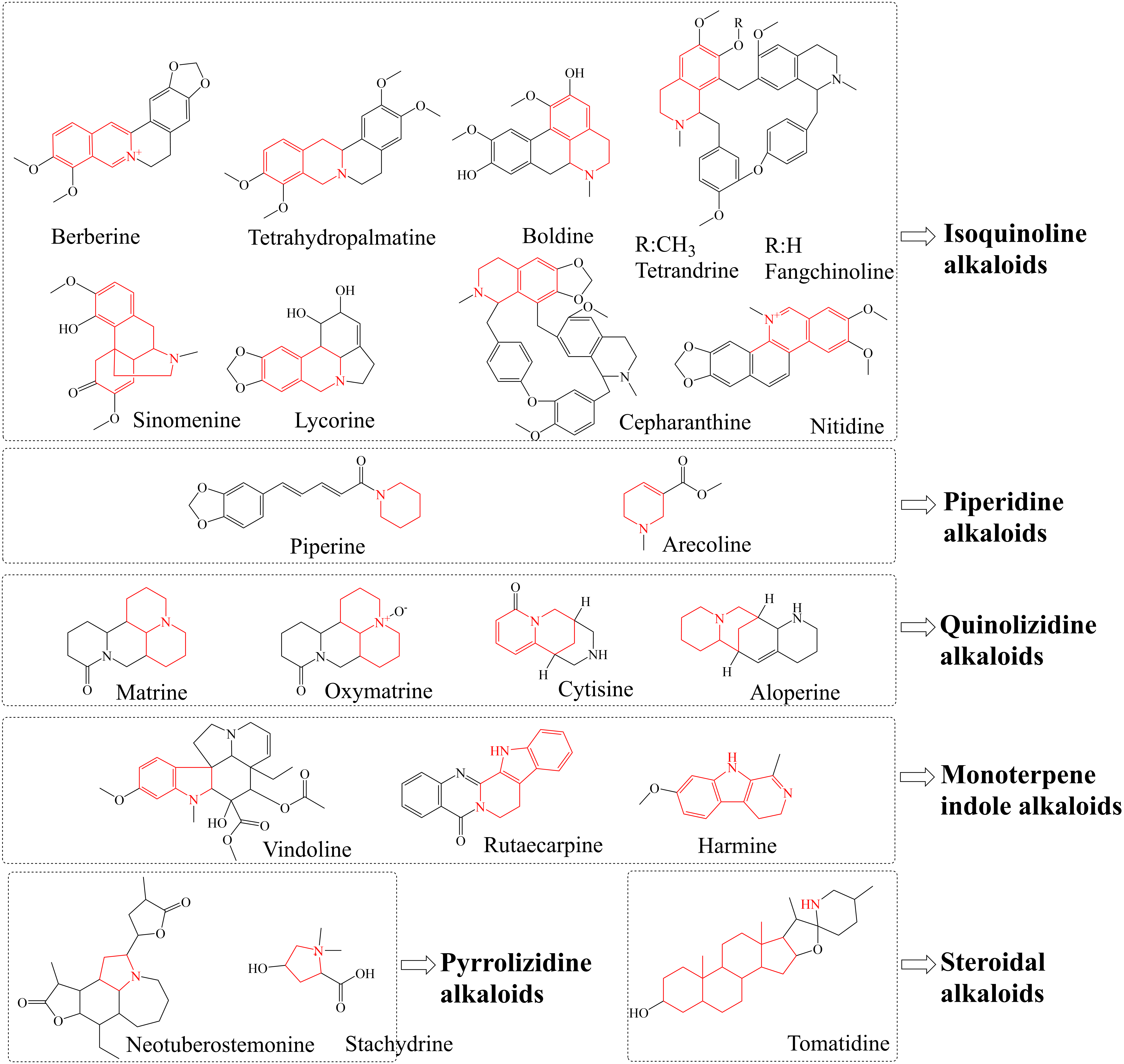

Supplement: Supplementary file 1 [file Image1.TIF]
